# A Novel *μ*CT Analysis Reveals Different Responses of Bioerosion and Secondary Accretion to Environmental Variability

**DOI:** 10.1371/journal.pone.0153058

**Published:** 2016-04-13

**Authors:** Nyssa J. Silbiger, Òscar Guadayol, Florence I. M. Thomas, Megan J. Donahue

**Affiliations:** 1 Department of Ecology and Evolutionary Biology, University of California at Irvine, Irvine, California, United States of America; 2 Hawai’i Institute of Marine Biology, University of Hawai’i at Mānoa, Kāne’ohe, Hawai’i, United States of America; 3 School of Life Sciences, Joseph Banks Laboratories, University of Lincoln, Lincoln, United Kingdom; James Cook University, AUSTRALIA

## Abstract

Corals build reefs through accretion of calcium carbonate (CaCO_3_) skeletons, but net reef growth also depends on bioerosion by grazers and borers and on secondary calcification by crustose coralline algae and other calcifying invertebrates. However, traditional field methods for quantifying secondary accretion and bioerosion confound both processes, do not measure them on the same time-scale, or are restricted to 2D methods. In a prior study, we compared multiple environmental drivers of net erosion using pre- and post-deployment micro-computed tomography scans (*μ*CT; calculated as the % change in volume of experimental CaCO_3_ blocks) and found a shift from net accretion to net erosion with increasing ocean acidity. Here, we present a novel *μ*CT method and detail a procedure that aligns and digitally subtracts pre- and post-deployment *μ*CT scans and measures the simultaneous response of secondary accretion and bioerosion on blocks exposed to the same environmental variation over the same time-scale. We tested our method on a dataset from a prior study and show that it can be used to uncover information previously unattainable using traditional methods. We demonstrated that secondary accretion and bioerosion are driven by different environmental parameters, bioerosion is more sensitive to ocean acidity than secondary accretion, and net erosion is driven more by changes in bioerosion than secondary accretion.

## Introduction

**Fig 1:**
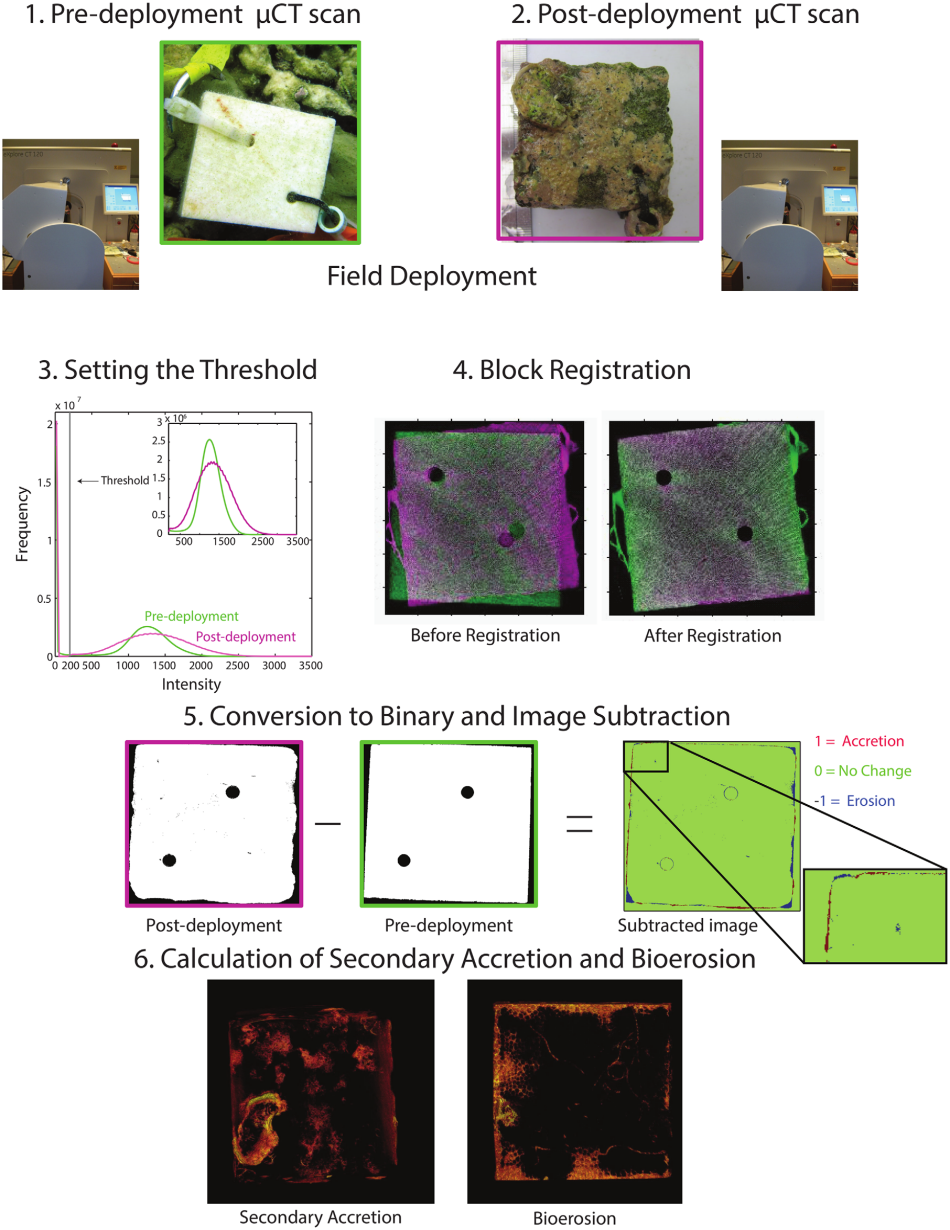
Schematic illustrating the *μ*CT methods. (1) Experimental blocks were cut from dead massive *Porites* spp. skeleton and sent to the Cornell University Multiscale CT facility for Imaging and Preclinical Research for pre-deployment scans. Blocks were scanned at a resolution of 50 *μ*m^3^ and then averaged to 100 *μ*m^3^ for data analysis. (2) Pre-scanned blocks were deployed along the reef transect for one year, retrieved, and scanned a second time. (3) During data analysis a threshold of 200 Hounsfield Units (shown by the grey line) was set to remove edge effects and separate CaCO_3_ fom air. Figure shows histograms for a pre-deployment block (green) and a post-deployment block (magenta). The inset shows the histograms of the blocks after thresholding. (4) Pre and post-deployment scans were aligned using image registration tools in MATLAB’s Image Processing Toolbox. Images are pre and post-deployment scans overlayed on top of each other before (left) and after (right) image registration. (5) Images were converted to binary (white is a value of 1 and black is a value of 0) and subtracted from each other. All positive values (red) were new pixels and were counted as secondary accretion and all negative values (blue) were lost pixels and counted as bioerosion. Values of zero (green) correspond to areas where there were no changes between pre and post-deployment scans. (6) We calculated secondary accretion by summing all positive values and bioerosion by summing all negative values in the subtracted image. Images are 3D representations highlighting only secondary accretion (left) and bioerosion (right). See supporting information for 3D movies of secondary accretion (S1 Movie) and bioerosion (S2 Movie) from the same experimental block. Image credits: N. Silbiger and M. Riccio.

Human-induced changes in ocean chemistry [1–9], temperature [1, 5, 9, 10], and water quality [3, 11–16] are threatening coral reefs [1, 11, 17]. Predictions of reef response to changing ocean conditions are often based on the response of reef building corals alone [17, 18]; however, coral reef bioerosion from borers (e.g., boring bivalves, sponges, and marine worms) and grazers (e.g., parrotfish and urchins) and secondary accretion from crustose coralline algae (CCA) and other encrusting invertebrates are also critical processes for reef sustainability [19]. Recent studies highlighting the sensitivity of bioerosion and secondary accretion to ocean acidification [3–8, 20] have sparked an interest to further investigate how these processes respond to natural variation and climate change stressors. Although there are currently a diverse set of field methods used to calculate reef accretion and bioerosion (reviewed in Table 1), few methods are available to simultaneously and separately measure accretion and bioerosion rates. For example, in prior studies, imaging methodologies in 2-dimensions (e.g., [21–23]) and, more recently, 3-dimensions (CT and *μ*CT) [3, 20, 24] have been applied to slabs or cores of reef to separate accretion and bioerosion (Table 1), but rates are difficult to estimate because the time the substrate became available to bioeroders and secondary calcifiers is unknown. Pre- and post-deployment buoyant weight [6, 8], volume [25], and mass [26–28] calculations on experimental substrates (Table 1) confound secondary accretion and bioerosion processes, but have a known deployment period and thus can be used to calculate a rate. Before and after *μ*CT scans can calculate net erosion and secondary accretion rates on experimental substrates [4], but also confound accretion and erosion processes. Calculating accurate accretion and erosion rates is dependent on the ability to separate accretion and erosion processes and to estimate the time-scale of each process. Here, we describe a new analysis using pre- and post-deployment *μ*CT scans to separate secondary accretion and bioerosion from the same experimental substrate exposed to the same environmental variation over the same time-scale (Fig 1) and, thus, allowing us to address how each of these processes independently respond to environmental stressors *in situ*. Our *μ*CT method also allows for a 3D visualization of the experimental substrates that highlights specific areas of secondary accretion and bioerosion (See S1 and S2 Movies in supporting information).

**Table 1:** Traditional Field Methods for Bioerosion Measurements: A table highlighting different methods published in the primary literature, a short description of each method, benefits and constraints of each method, and selected publications.

Method	Method Description	Benefits	Constraints	Publications
Change in weight, height, volume, or density of experimental block	Deploy blocks of CaCO_3_ on a reef for a set time and measure the difference in weight, height, volume, or density between the pre- and post-deployment blocks.	Calculates an accurate rate because the block deployment time is known Erosion rate is inclusive of both internal and external eroders	Measures a net change in the block and does not discriminate accretion and erosion processes Blocks need to be deployed for approx. 5 years to include late succesional stage eroders	[6, 8, 25, 26, 28–30]
Casts or Molds	Impregnate samples with epoxy resin and dissolve sample with dilute HCl. Results in 3D cast of bioerosion scars.	Separates accretion and erosion Visualize boring scars in 3D	Poor estimate of bioerosion rate because the actual time when CaCO_3_ becomes available is unknown	[31–33]
X-ray and other 2-dimensional image analyses	Collect live coral cores or dead coral rubble, cut the sample into slabs, and take a picture, X-ray, or trace erosion scars onto a piece of paper.	Separates accretion and erosion. Using reef samples, as opposed to experimental blocks, likely includes an advanced succesional stage of eroders and calcifiers	Poor estimate of bioerosion rate because the actual time when CaCO_3_ becomes available is unknown Results may under- or over-estimate erosion rates depending on where the slab was cut	[12–15, 21–23, 25, 34–50]
Count grazing scars by eroding fish	Track parrotfish, note when they remove CaCO_3_ from the reef, and measure volume of grazing scar.	Able to calculate grazing rates based on size or species of fish	Only accounts for parrotfish erosion	[51–53]
Count bore holes along a reef transect	Count bore holes from bieoroding animals on the surface of live or dead coral *in situ*.	Inexpensive and quick Includes counts of different types of macroborers	Only accounts for macroborers large enough to make a hole that is visible without magnification Poor estimate of bioerosion rate because the actual time when CaCO_3_ becomes available to borers is unknown	[16, 54, 55]
Scanning Electron Microscopy (SEM)	Millimeter sections of a sample are cut with a diamond blade saw, embedded with resin, etched with dilute HCl, and sometimes coated in platinum. Surface area bioeroded from each sample is quantified with 2D image analysis	Very high resolution images of microborings	Only accounts for microbioerosion Results are highly dependent on where cuts are made	[14, 21, 31–34, 42, 56–60]
Single CT or *μ*CT scan	Scan live or dead coral cores using a CT or *μ*CT scanner.	Separates accretion from erosion Visualizes erosion scars in 3D Calculates accretion from measuring coral annual density bands	Poor estimate of bioerosion rate because the actual time when CaCO_3_ becomes available to eroders is unknown	[3, 20, 24, 61–64]
Before and after *μ*CT scan	See methods section.	High resolution 3D measure of both accretion and erosion Visualizes boring scars in 3D Using before and after scans allows for the removal of any pre-existing boring scars Calculates an accurate rate since deployment time is known	Blocks need to be deployed for a long period of time to quantify late succesional stage bioeroders. Can be costly depending on resolution of scan	[4], Present Study

We tested a new method using a previously published dataset that used pre- and post-deployment *μ*CT scans to calculate net erosion rates along a natural gradient in Kāne‘ohe Bay, Hawai‘i. Like many reefs around the globe [24, 38, 65–67], Kāne‘ohe Bay has persistent areas of natural acidification that reach the low open-ocean pH values expected by the end of the 21st century [4, 68] that are likely driven by differences in tidal flushing, photosynthesis and respiration, ground-water inputs, and other benthic biological processes [67, 69–73]. Using a space-for-time framework, we can leverage this natural variability to understand how reefs will respond to ocean acidification in the context of a naturally variable environment. Our prior work in Kāne‘ohe Bay demonstrates that net reef erosion (calculated as the percent change in volume of experimental CaCO_3_ blocks) is driven by natural variation in pH and that reefs could shift from net accretion to net erosion with increasing ocean acidity [4], but the underlying mechanisms driving this shift are unknown. Here, we present a major advance to our prior method that allows us to separate secondary accretion and bioerosion rates from the same experimental substrate. Here, we leverage the data from our prior study [4] and, using this novel method, we uncover results that could not be obtained with any of the pre-existing methods.

## Materials and Methods

### Experimental Block Construction

Experimental blocks were cut from dead pieces of the massive coral *Porites* sp. skeleton into 5cm x 5cm x 2cm blocks with a diamond blade rock saw. Blocks were carefully inspected and any blocks with obviously pre-existing boreholes were discarded. Blocks were then soaked in freshwater and autoclaved to sterilize the substrate. Two holes were drilled into each block for cable ties in order to attach the blocks to the reef. The average skeletal density of the experimental blocks, measured using the buoyant weight technique, were 1.57 ±0.07(sd) (n = 21) g cm^−3^.

### *μ*CT Analysis

Secondary accretion and bioerosion rates were calculated using *μ*CT (Fig 1). *μ*CT is an X-ray technology that non-destructively images the external and internal structures of solid objects, resulting in a three-dimensional array of object densities. We used an eXplore CT120 *μ*CT (GE Healthcare Xradia, Inc) at the Cornell University Imaging Multiscale CT Facility (Fig 1.1 and 1.2) to scan blocks before and after deployment (voltage = 100kV, current = 50mA). Angular projections were acquired in a full 360° rotation in 0.5° increments; two images at each angle were acquired and averaged creating a three-dimensional array of isotropic voxels at 50 *μ*m^3^ which was then averaged to 100 *μ*m^3^. Data were stored as .vff files and were transferred to a MacPro (2 × 2.26 GHz quad-core Intel Xeon, 32 GB, 1066 MHz DDR3) for data analysis. An intensity value of 200 was set as a global threshold to separate CaCO_3_ from air and remove any effects of partial volume averaging at the coral block-air interface [4] (Fig 1.3). Intensity values are directly correlated with skeletal density at each pixel. The number of voxels exceeding this threshold was used in calculating secondary accretion and bioerosion. Pre- and post-deployment scans were aligned, or registered, using an intensity-based image registration algorithm from the MATLAB ^®^Image Processing Toolbox (Fig 1.4). We used the One Plus One Evolutionary Optimizer, an iterative algorithm that maximizes the best registration results by perturbing the parameters between iterations [74], as our optimization technique. Mattes Mutual Information metric maximizes the number of corresponding pixels with similar intensity values [75] which was used to describe the accuracy of the registration. After the images were registered, both pre- and post-deployment scans were converted to binary, such that any positive intensity value (a pixel with CaCO_3_) was assigned a one and all other values (air) were assigned a zero (Fig 1.5). The two images were then subtracted from one another giving a matrix of 1’s, 0’s, and −1’s. In the subtracted matrix, all pixels with a value of one represented areas of new CaCO_3_ (accretion) and all values of negative one were areas where CaCO_3_ was removed (bioerosion) (Fig 1.6). A value of zero meant there was no change at that pixel between the before and after scans. Converting images to binary is the most conservative way to calculate secondary accretion and bioerosion; it does not account for any change in skeletal density, but rather an absolute loss of CaCO_3_. Subtracting the two raw images, without converting to binary, would potentially over-estimate secondary accretion and bioerosion due to partial volume averaging of surrounding pixels or a change in skeletal density due to chemical dissolution.

To calculate secondary accretion and bioerosion rates, all positive and negative values were summed in the subtracted matrix and multiplied by the voxel size (100 *μ*m)^3^ to give the total volume of CaCO_3_ gained or lost, respectively. Bioerosion and secondary accretion rates were calculated using the following equations: Bioerosion Rate (kg m^−2^ yr^−1^) = (*Vol*_*i*_ × *ρ*_*i*_)/(*SA*_*i*_ × *Time*) and Secondary Accretion Rate (mm yr^−1^) = *Vol*_*i*_/(*SA*_*i*_ × *Time*), where *i* represents an individual block, *Vol* is the volume lost (bioerosion) or gained (secondary accretion) in m^3^, *SA* is the surface area of the pre-deployment blocks (m^2^), *ρ* is the skeletal density of the pre-deployment block (kg m^−3^), and *Time* is the deployment time (years) on the reef. Secondary accretion rates were converted from m to mm per year to compare with literature values. Bulk skeletal density and volume of pre-deployment blocks were calculated using the buoyant weight technique on a Mettler-Toledo balance (accuracy of 0.01 mg). Surface area was calculated from the images following methods by [76].

### Accuracy

To test the accuracy of the volumes calculated using *μ*CT, buoyant weight and *μ*CT calculated volumes were compared to each other from the pre-deployment blocks with a simple linear regression. Accuracy of volume calculated using the *μ*CT method was determined by the *R*^2^ value of the linear regression.

### Experimental Design

The experimental design is described in a previously published study [4], which we summarize here (see supporting information for full description of experiment; S1 Methods). Patterns in carbonate chemistry, nutrients, chlorophyll *a*, temperature, and depth were characterized along a 32 m transect (S1 Fig; described in Silbiger et al. (2014)), and used to compare five specific hypotheses about drivers of the accretion-erosion balance: carbonate chemistry, resource availability, temperature, depth, and hydrodynamics (distance from shore and depth). We used a model selection approach to test which of these drivers has the strongest relationship to secondary accretion and bioerosion rates calculated with our novel *μ*CT analysis.

Our study site was located in Kāne‘ohe Bay, O‘ahu on the windward (eastern) side of Moku o Lo‘e. Twenty-one experimental blocks were deployed along a 32 m transect, stratified between reef flat and reef slope (S2 Fig). Field deployments and collections were made under special activity permit # SAP2011-1 to the Hawai’i Institute of Marine Biology at the University of Hawai’i at Mānoa. Blocks were deployed from March 31, 2011 to April 10, 2012. We collected both discrete water samples (pH, TA, nitrate, nitrite, ammonium, phosphate, and chlorophyll *a*) and data from continuous sensors (temperature and depth) along the transect. Water samples were collected directly above each block four times within 24 hours in September, December, and April in order to capture both diel and seasonal variability in the environment. Continuous sensors were stationed over each block for a minimum of two weeks to calculate high frequency (0.1 min^−1^) variation in temperature and depth. These short time series were normalized to a continuous time series from a permanent station positioned adjacent to the transect, allowing comparison of the micro-environments at each block [68].

### Model Selection

Our goal was to compare known drivers and correlates of the accretion-erosion balance. Many of the environmental variables were collinear along the transect; thus, we removed collinearity by using the residuals of a regression of each environmental variable against depth and distance from shore [4]. Correlation coefficients for raw environmental data and the residual environmental data are available in Silbiger et al. (2014) [4].

We used a model selection framework to compare models for five specific hypotheses about the accretion-erosion balance, test which of these drivers had the strongest relationship to secondary accretion and bioerosion (Table 2), and compared those results to net erosion rates from Silbiger et al. (2014) [4]. In a model selection framework, Akaike Information Criterion (AIC) values are used to rank candidate models, accounting for both fit and complexity. Carefully constructed model selection avoids problems associated with multiple hypothesis testing that are common in stepwise regression, such as arbitrary *α* levels and uninterpretable functional relationships [77, 78]. Here, we used the corrected AIC (AICc), which is recommended for sample sizes <30 [78]. While the model with the smallest AICc value (ΔAICc = 0) is the ‘best’ of the models considered, models with an ΔAICc value of <4 have some empirical support [78]. The five models were: carbonate chemistry, resource availability, temperature, depth and distance and full model. We used pH to test how carbonate chemistry influenced secondary accretion, bioerosion, and net erosion rates. Carbonate chemistry parameters are inherently correlated, and pH had the strongest relationship of the carbonate chemistry parameters (S1 Table). The pH model includes both the mean and variance of the discrete pH samples from each block. The resource availability model includes the means and variances of DIN:DIP ratios (a proxy for resource quality) and chlorophyll *a* (a measure of resource quantity) from the discrete water samples. The temperature model included the mean relative temperature anomaly of each block from the overlaying water column and temperature covariance between the block and overlaying water column. The final models were of depth and distance from shore. These linear models were compared to a full model that includes the means and variances of every parameter stated above (Table 2). Environmental data that did not meet the assumptions of normality were log-transformed (mean Chlorophyll *a* and mean DIN:DIP), secondary accretion, bioerosion, and net erosion data were square-root transformed to meet assumptions of normality, and one block with a large aggregation of oysters was excluded from the analysis (n = 20 blocks in the analysis). Figures showing secondary accretion (S3 Fig) and bioerosion (S4 Fig) versus the means and variances of all environmental parameters are available in the supporting information and figures showing net erosion versus all environmental parameters are available in Silbiger et al. (2014) [4].

**Table 2:** Model Selection for (a) bioerosion and (b) secondary accretion versus environmental parameters.

Model Parameters	k	-log(L)	AICc	ΔAIC	R^2^	Rank
***(a) Model selection for bioerosion vs environmental parameters***						
**pH**	4	-12.54	-17.58	0	0.50	1
$Y∼\bar{pH}+Var\left(pH\right)$						
**Distance**	3	-5.93	-7.15	10.43	0.04	2
*Y* ∼ *Distance*						
**Depth**	3	-5.54	-6.38	11.20	0.004	3
$Y∼\bar{Depth}$						
**Resource Availability**	6	-10.17	-6.05	11.53	0.37	4
$Y∼\bar{Chl}+Var\left(Chl\right)+\bar{DIN:DIP}+Var\left(DIN:DIP\right)$						
**Temperature**	4	-5.94	-4.37	13.21	0.04	5
$Y∼\bar{Temp}+Covar\left(Temp\right)$						
**Full**	12	-22.87	9.26	26.84	0.82	6
$\begin{array}{l} Y∼\bar{pH}+Var\left(pH\right)+\bar{Temp}+Covar\left(Temp\right)+\bar{Chl}+\\ Var\left(Chl\right)+\bar{DIN:DIP}+Var\left(DIN:DIP\right)+\bar{Depth}+\\ Distance \end{array}$						
***(b) Model selection for secondary accretion vs environmental parameters***						
**Distance**	3	-16.29	-27.87	0	0.23	1
**Depth**	3	-15.10	-25.49	2.38	0.13	2
**pH**	4	-15.05	-22.60	5.27	0.12	3
**Temperature**	4	-14.45	-21.40	6.47	0.07	4
**Full**	12	-36.25	-17.50	10.38	0.89	5
**Resource Availability**	6	-14.62	-14.94	12.93	0.09	6

k is the number of parameters in the model, -log(L) is the negative log likelihood of the model, *AIC*_*c*_ is the Akaike Information Criterion corrected, Δ*AIC*_*c*_ is the difference from the lowest *AIC*_*c*_ value, *R*^2^ is the proportion of total variance explained by the model, and Rank is the rank of the model with 1 representing the best fit. Each model is a linear regression of bioerosion or secondary accretion versus the means ($\bar{X}$) and variances (*Var*(*X*)) or covariance (*Cov*(*X*)) of each parameter. The Resource Availability Model includes DIN:DIP and chlorophyll a concentration and the Full Model includes means and variances (or, for temperature anomaly, covariance) for all environmental parameters. Environmental data are the residuals from a regression between each parameter versus depth and distance from shore. Secondary accretion and bioerosion rates were square-root transformed to meet model assumptions. The upper table is the model selection for bioerosion and the lower table is the model selection for secondary accretion.

We calculated standardized regression coefficients for the highest ranking models for secondary accretion, bioerosion, and net erosion to determine the relative effect size of the models. Standardized regression coefficients were calculated by z-scoring both the environmental data and the accretion-erosion rates, re-calculating the linear regression models with the standardized data, and taking the absolute value of the coefficients from each model. The standardized regression coefficients allow for comparison within a rate between the environmental variables as well as across rates within a single variable.

## Assessment

### Accuracy

**Fig 2:**
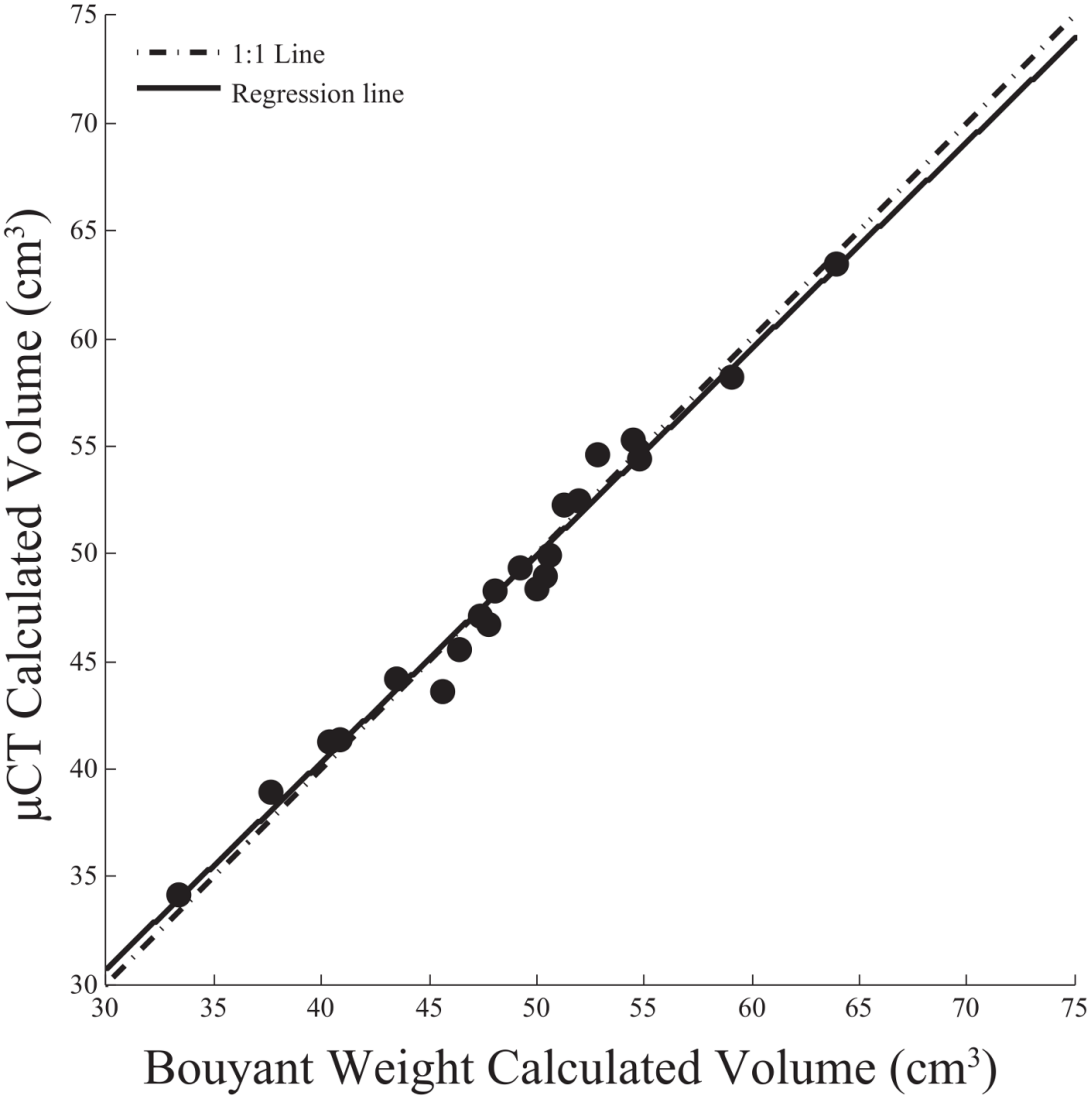
Comparison of calculated volumes (*cm*^3^) using the buoyant weight and *μ*CT methods described in this paper. Black circles are volumes calculated from the pre-deployment experimental blocks. We used a linear regression to test the relationship between the buoyant weight and *μ*CT methods. The solid black line is the best fit line from the regression and the dashed line is a 1:1 line. The pre-deployment volumes calculated from each method are highly co-linear (F_19_ = 859, p<0.001, R = 0.98, *y* = 0.96*x* + 1.9).

We compared the volume of the pre-deployment blocks calculated with *μ*CT (volume was calculated by summing the number of voxels that exceeded the threshold and multiplying the sum by the voxel size (Fig 1)) to the volume calculated using buoyant weight and the data are in close agreement: the volumes calculated from *μ*CT are nearly identical to standard buoyant weight methods (Fig 2; F_19_ = 859, p<0.001, R^2^ = 0.98, *y* = 0.96*x* + 1.9) indicating that *μ*CT provides an accurate representation of the block volume.

### Time and Cost

The scanning and 3D reconstruction of each experimental block with the GE Console Software took approximately 45 minutes. The registration technique in MATLAB took anywhere from 5 minutes to 2 hours per block and depended on the difference in orientation between the pre- and post-deployment blocks during scanning. Blocks that were positioned differently during post-deployment took much longer to register. Each scan cost $100 at the Cornell University Imaging Multiscale CT Facility for a total of $200 per block (pre- and post-deployment scans).

## Results and Discussion

### Bioerosion Rates

**Fig 3:**
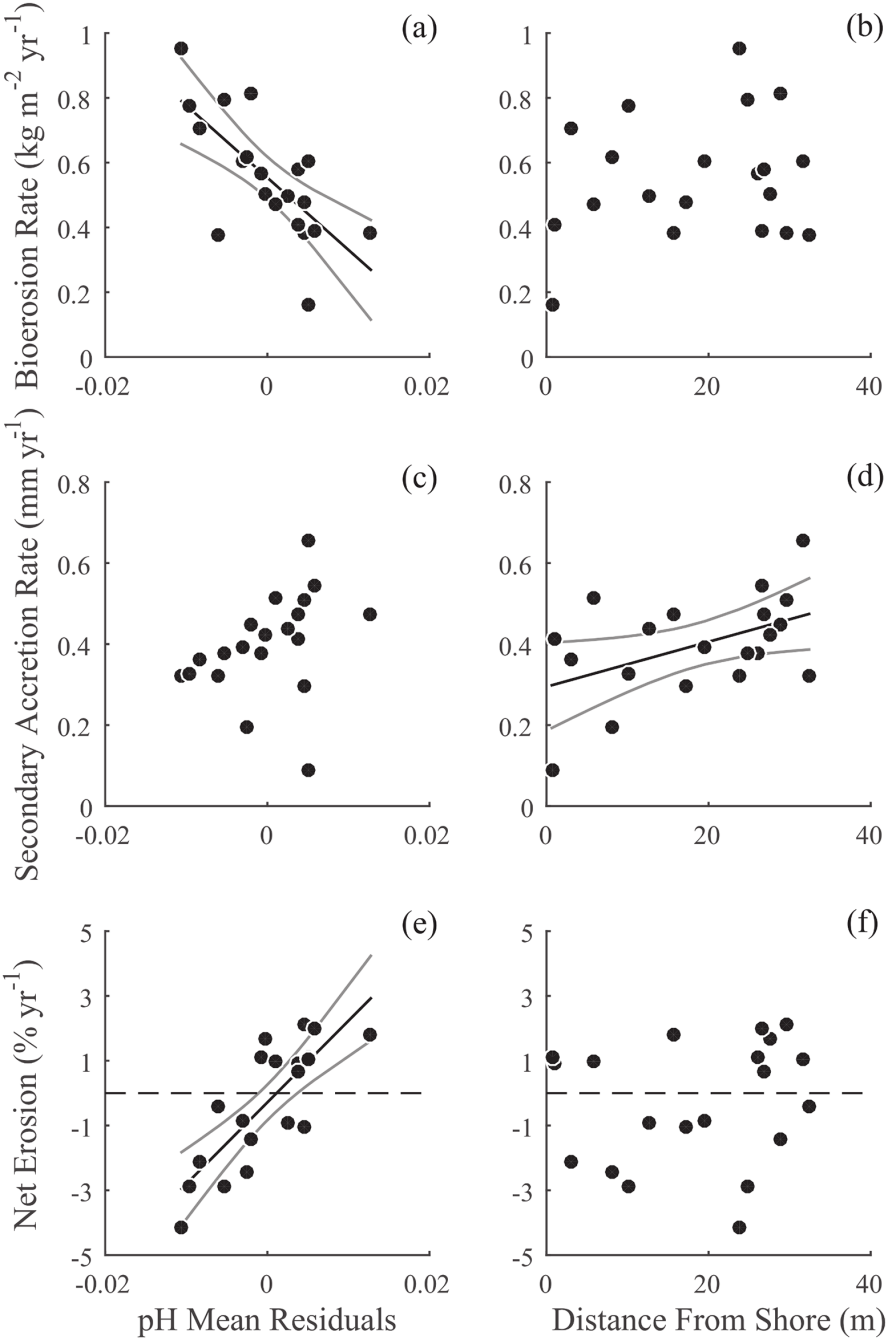
pH and distance from shore versus bioerosion, secondary accretion, and net erosion. Scatter plots for (a, b) bioerosion (kg CaCO_3_ m^−2^ yr^−1^), (c, d) secondary accretion (mm CaCO_3_ yr^−1^), and (e, f) net erosion (%Change in Volume yr^−1^) rates of experimental blocks (N = 20) versus (a, c, e) pH mean residuals (the top ranking model for bioerosion and net erosion) and (b, d, f) distance from shore (the top ranking model for accretion). Panels showing net erosion are from Silbiger et al (2014) [4]. Best fit model and 95% confidence intervals are shown for the highest ranking model for each rate (Table 2): bioerosion vs pH mean residuals (*y* = −22.29*x* + 0.55, *R*^2^ = 0.50), secondary accretion vs distance from shore (*y* = 5.54*E* − 3*x* + 0.29, *R*^2^ = 0.23), and net erosion vs pH mean residuals (*y* = 251.81*x* − 0.29, *R*^2^ = 0.64). The standardized regression coefficients for each of these models are shown in Fig 4. Dashed lines in panels e-f show where the blocks switch from net accretion to net erosion. pH mean was regressed against depth and distance from shore, and the residuals were used in the analysis and this figure. All rates were square-root transformed to meet model assumptions.

Bioerosion rates varied by more than an order of magnitude across our 32 m transect, ranging from 0.02 to 0.91 kg m^−2^ yr^−1^ (S4p Fig). These bioerosion rates are similar to rates at other Pacific reefs sites: a recent study using single CT scans from cores of live reef found bioerosion rates ranging from 0 to 0.6 kg m^−2^ yr^−1^ at remote reefs across the Pacific [3]. Interestingly, the range of bioerosion rates on our transect was greater than the range of bioerosion rates in this Pacific wide study, highlighting the importance of small-scale, within-reef variability. Bioerosion rates were best predicted by the pH model, which explained 50% of the variance in bioerosion across the transect (Table 2a, Fig 3, S5 Fig). The second best model, the distance model, had low empirical support (Δ*AIC*_*c*_ = 10.43) and explained only 4% of the variance (Table 2a). While the resource availability model described 37% of the variance in the data, it also had a larger number of parameters (6, including mean and variance for both DIN:DIP and chlorophyll *a*) and, therefore, ranked fourth in model parsimony. The full model, which included the means and variances of all parameters, described 82% of the variance in bioerosion rates indicating that the environmental data we collected adequately described patterns in bioerosion rates across the transect. Any additional environmental parameter would at most only explain 18% of the variance in bioerosion.

While all the parameters in these models interact to drive patterns in bioerosion, a ranking of individual parameters indicates that pH was the dominant driver. It is becoming clear that ocean acidity facilitates erosion [4–9], but the mechanisms that control this relationship are still not well known. Several studies suggest that ocean acidification could enhance chemical erosion (e.g., [5, 6, 8]) because many bioeroders erode the coral skeleton by secreting acidic compounds [79]. Lower pH in the overlaying water-column might make it metabolically easier for the bioeroders to reduce pH at the site of erosion and therefore promote erosion.

### Secondary Accretion Rates

Secondary accretion rates ranged from 0.01 to 0.4 mm yr^−1^ across the transect (S3p Fig). These rates are slightly lower than secondary accretion rates from a Kāne’ohe Bay study that saw 2 mm crusts of CCA after a 6 mo. exposure, perhaps due to differences in grazing between study sites or the size of the experimental blocks [80]. For secondary accretion, pH was not the best predictor for patterns in accretion across the transect (*R*^2^ = 0.12; Table 2b). Rather, the distance from shore model ranked highest explaining 23% of the variance in the data (Fig 3d) followed by the depth model explaining 13% of the variance. Differences in light and hydrodynamics along the transect could be mediating the relationship between secondary accretion and distance from shore and depth. Notably, our accretion rates were limited to secondary calcifiers such as CCA and encrusting invertebrates (e.g., oysters and barnacles), and excluded measurements of coral growth from adult corals. We did not measure light or photosynthetically active radiation across our transect, but our deepest site was only 4.5m deep, and, therefore, it is unlikely that light limited CCA growth across the transect. Further, distance from shore explained more of the variation in secondary accretion than depth (23% vs 13%; Fig 3d), and there is a tight correlation between distance from shore and turbulent kinetic energy dissipation rate (*R*^2^ = 0.88, S6 Fig), suggesting that hydrodynamics may be driving the patterns in accretion. Hydrodynamic energy (e.g., turbulence, wave action, tidal mixing) could impact secondary accretion in several ways: 1) both the delivery of dissolved compounds and particulates are positively correlated with hydrodynamic parameters increasing nutrient availability for benthic organisms [81, 82], 2) increased flow could promote accretion by facilitating settlement of benthic invertebrate larval recruits, such as oysters and barnacles [83], and 3) different exchange, or mixing, rates with offshore waters could impact accretion by replenishing food supplies and removing waste [84]. On our reef transect, the furthest offshore sites on the reef slope were constantly mixed with offshore deep water masses, whereas the water inside the reef flat was sometimes isolated. Therefore, large-scale mixing is a likely mechanism driving the patterns between accretion and distance from shore. Although pH ranked lower than distance from shore here, other manipulative studies have shown that changing pH can inhibit growth, abundance, and calcification rates of secondary calcifiers [85–87]. Lastly, the full model explained 89% of the variance in secondary accretion. Again, this indicates that the measured parameters adequately described patterns in secondary accretion. Any additional parameter would only add at most 11% explanatory power to the over-all model.

### Bioerosion and Secondary Accretion vs Net Erosion from Silbiger et al. (2014)

**Fig 4:**
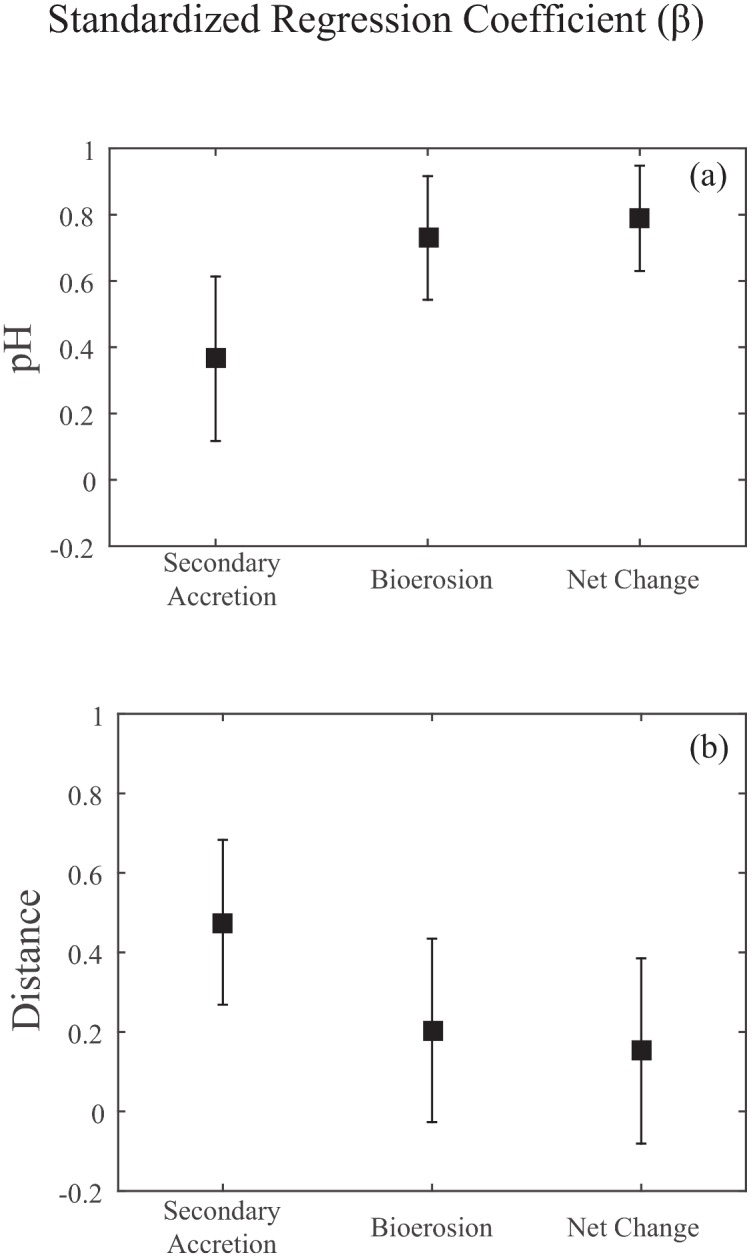
Standardized regression coefficients for secondary accretion, bioerosion, and net erosion rates vs (a) pH mean and (b) distance from shore. Squares are the standardized regression coefficients for each rate versus distance from shore and the partial standardized regression coefficients for each rate versus pH mean (partial coefficients because the pH model included both mean and variance). Error bars are standard errors of the mean.

In a prior study, we saw a shift from net accretion to net erosion with increasing ocean acidity [4], but we were unable to uncover the underlying mechanisms driving this shift. By separating secondary accretion and bioerosion processes we demonstrated that these processes are driven by different environmental parameters: pH was the highest ranking model for bioerosion while distance from shore was the highest ranking model for secondary accretion (Table 2). Indeed, accretion and erosion rates on coral reefs are controlled by different organisms, so it is not surprising that they respond to different environmental parameters. Yet, this is the first method to simultaneously measure secondary accretion and bioerosion on the same time-scale and compare multiple drivers of the accretion-erosion balance. This new analysis also indicates that bioerosion is more sensitive to ocean acidity than secondary accretion. The proportion of variance explained (*R*^2^ = 0.50 vs 0.12; Table 2) and the effect size in the pH model was higher for bioerosion than for secondary accretion: for a 0.1 increase in pH, we saw a 73% (±18% SE) decrease in bioerosion compared to a 37% (±25% SE) increase in secondary accretion (Figs 3a, 3c and 4a), indicating that bioerosion responded more strongly to pH than secondary accretion. Bioerosion also responded to pH mean 14.4 times more strongly than pH variance. The highest ranking model for secondary accretion was distance from shore (Table 2b). Secondary accretion increased by 48% (±20% SE) while bioerosion only increased by 20% (±23% SE) per m from shore (Figs 3b, 3d and 4b). Further, the highest ranking model for net erosion from Silbiger et al. (2014) [4] was pH: the pH model had an *R*^2^ of 0.64 (Table 1 from [4]) and there was a 79% (±16% SE) decrease in net erosion (or, increase in net accretion) for every 0.1 pH unit increase (Figs 3e and 4a). There was only a 15% (± 23% SE) decrease in net erosion (or, increase in net accretion) with distance from shore (Figs 3f and 4b). The similar responses of net erosion and bioerosion to the environmental drivers indicate that the bioerosion response is driving the shift from net accretion to net erosion.

Our data indicate that reef erosion (and dissolution), rather than reef accretion, may be driving the negative relationship between ocean acidification and net calcification of coral reefs. This has significant implications for coral reef predictions. Recent studies support this hypothesis [5, 88, 89]: in a laboratory experiment, chemical dissolution from bioeroders was more strongly correlated with ocean acidity than was secondary calcification [5], and in a field study, live coral and mollusc calcification was unaffected by natural acidification at CO_2_ vents in the Mediterranean at normal temperatures, but dissolution of dead skeletons increased with decreasing pH [88]. Here, we demonstrate that bioerosion is more sensitive to ocean acidity than secondary accretion along a natural environmental gradient. Our results and those from previous studies [3–7, 9] provide compelling evidence that erosion rates will increase under future ocean conditions. The sensitivity of erosion to ocean acidification could tip the balance of coral reefs in favor of net reef erosion in a more acidic ocean.

### Advancing Methods for Examining Secondary Accretion and Bioerosion

To predict how reefs may shift in the future, it is necessary to understand how accretion and erosion processes respond to environmental variation. Yet, prior methods that analyze accretion and erosion confound both processes, do not measure them on the same time-scale, or are restricted to 2D methods (Table 1). In a prior study, we used before and after *μ*CT scans to calculate the net change in volume of experimental CaCO_3_ blocks [4]. In the present study, we advance this method by aligning and differencing before and after scans to separate changes due to secondary accretion and bioerosion. *μ*CT can be used to calculate how much volume is added or removed from an experimental block to accuracy determined by the resolution of the scan (here, 100 *μ*m). When comparing *μ*CT and buoyant weight calculated volumes to each other the volumes were nearly identical (Fig 2), but *μ*CT provides a more complete analysis of secondary accretion and bioerosion processes. Further, prior studies that used experimental substrates had to meticulously examine the substrate for pre-existing boreholes because the presence of boreholes can bias the analysis. Using before and after *μ*CT and subtracting the two images accounts for and digitally removes the effect of any pre-existing borings, allowing for the use of more realistic reef substrates.

Uncovering the mechanisms driving the shift from net accretion to net erosion was only possible with our new *μ*CT analysis; using this method has the potential to expose several gaps in our knowledge about the response of coral reefs to future ocean conditions and to answer whether reef accretion will continue to exceed reef erosion. For example, will primary accretion, secondary accretion, and reef erosion respond similarly to environmental drivers and will their responses combine to accelerate reef loss? Studies that examine accretion or erosion processes individually have found different responses to environmental stress. In a field experiment in Indonesia, coral calcification and bioerosion had different functional relationships with land-based pollution [15]. Laboratory experiments focusing on climate stressors (i.e. temperature and ocean acidity) have found that bioerosion is linearly related to ocean acidity and temperature [5–9], but that calcification exhibits both linear [17, 86] and non-linear [5, 17, 86, 90] responses. These differential responses of primary and secondary accretion and bioerosion challenge our ability to predict the net response of coral reefs to environmental change. Further, how will multiple environmental stressors impact individual reef processes? Many environmental parameters interact to drive patterns in accretion and erosion, including ocean acidity [1–9], temperature [1, 5, 9, 10], nutrients [3, 11–14], and gradients of human influence (e.g., chlorophyll, turbidity, sedimentation) [15, 16]. This myriad of drivers complicates the predictions of reef response to climate change. Using *μ*CT we can separate accretion and erosion processes and determine how different drivers influence each process independently. In this study, we saw that bioerosion was driven by changes in ocean acidity while secondary accretion was driven by changes in hydrodynamics along our local gradient and that net erosion is driven more by changes in bioerosion than secondary accretion. Using *μ*CT to calculate secondary accretion and bioerosion will improve our ability to predict how coral reefs will respond to a changing environment.

## Conclusions

### Comments and Recommendations

There are two disadvantages to this method: 1) it does not include a measurement for primary accretion from corals and 2) it can be costly. Although corals are not included in our analysis, this is the first method that can simultaneously measure secondary accretion and erosion from the same substrate in 3D and would complement studies that aim to also measure primary accretion. While before and after *μ*CT scans do cost more than single CT scans or before and after weights using traditional methods, there is far more information that can be gleaned from this method than traditional methods. In addition to separating secondary accretion and bioerosion from the same substrate, we can also visualize boring scars and settlement of accreters in 3D (See S1 and S2 Movies). These visualizations can be used to extract information about the organisms involved in secondary accretion and bioerosion. The cost of the scanning is directly related to the size of the experimental block and the resolution of the scan. We used a 50 *μ*m resolution which we averaged to a 100 *μ*m for data analysis (50 *μ*m was too high for a standard computer during alignment). Note that this volumetric analysis measures changes at the voxel scale of 100 *μ*m^3^, and, therefore, may underestimate bioerosion by microborers, which make erosion scars between 1 and 100 *μ*m [56]. A user interested only in macroborers can minimize the costs by reducing the resolution of the scan, whereas one interested in including microeroders in the analysis will need a higher resolution, which is possible to the sub-micron level with *μ* or nanoCT. Additionally, it is important to note that the substrate type used to construct the experimental block can influence bioerosion rates [37, 91]. For example, a 9 mo. study that examined sponge bioerosion rates on eight different coral species found that massive *Porites* species (similar to the blocks used here) had an erosion rate that was 2.8 times higher than *Astreopora listeri* [91]. It would be interesting to use this *μ*CT method to test if the relationship between environmental drivers and secondary accretion-bioerosion rates persist across different substrate types (i.e. coral species). Lastly, we offer a recommendation to those interested in using this method. During pre- and post-deployment scans we simply placed the blocks directly into the *μ*CT scanner for analysis. We recommend that blocks be scanned in a holder so that the orientation of the blocks is exactly the same for each scan. This ensures a much faster registration during post-processing. As bioerosion is now being included as a monitoring tool on coral reefs throughout the Pacific and the Atlantic (pers com. Dr. Rusty Brainard, National Oceanic and Atmospheric Administration Coral Reef Ecosystem Division), this new tool will provide comparable estimates of bioerosion and secondary accretion rates between sites and over long time scales.

## Supporting Information

S1 Movie 3D visualization of *μ*CT scan highlighting secondary accretion onto a block (same block as S2 Movie). (MOV)

S2 Movie 3D visualization of *μ*CT scan highlighting bioerosion from a block (same block as S1 Movie). (MOV)

S1 Fig Environmental data. Means and variances for temperature anomalies (a-b), chlorophyll *a* (*μ*g l^−1^) (c-d), DIN:DIP (e-f), and pH_*t*_ (total scale) (g-h) along the transect (N = 21).
(PDF)

S2 Fig Schematic of reef transect. Experimental blocks (grey rectangles) were stratified between reef flat and reef slope along a 32 m transect and were deployed for one year. The average depth ranged from 0.5 to 4.5 m. Discrete environmental samples were collected directly above each experimental block. Continuous sensors were stationed over each block for a minimum of two weeks (mobile sensors) and were normalized to a continuous time series from a permanent sensor station (Permanent sensors). Picture of YSI Sonde is from sontek.com.
(PDF)

S3 Fig Secondary accretion versus the means and variances of all environmental parameters. Environmental parameters were regressed against depth and distance from shore and the residuals from those regressions are used in this figure.
(PDF)

S4 Fig Bioerosion versus the means and variances of all environmental parameters. Environmental parameters were regressed against depth and distance from shore and the residuals from those regressions are used in this figure.
(PDF)

S5 Fig All discrete pH samples from September, December, and April sampling periods across the transect. In each sampling period, water samples were collected at 08H00 (blue), 14H00 (green), 20H00 (black), and 02H00 (magenta), resulting in 12 samples at each of the 21 blocks.
(PDF)

S6 Fig Turbulent kinetic energy dissipation rate (*ϵ*) (*m*^2^ *s*^−3^) versus distance from shore (n = 11). Turbulence was measured at 11 of the 21 sites and there was a significant relationship between *ϵ* and distance from shore (*F*_11,9_ = 63.1, p<0.0001, *R*^2^ = 0.88).
(PDF)

S1 TableBioerosion Model Selection with all carbonate parameters.k is the number of parameters in the model, -log(L) is the negative log likelihood of the model, *AIC*_*c*_ is the Akaike Information Criterion corrected, Δ*AIC*_*c*_ is the difference from the lowest *AIC*_*c*_ value, *R*^2^ is the proportion of total variance explained by the model, and Rank is the rank of the model with 1 representing the best fit. Each model is a linear regression of total bioerosion versus the means $\left(\bar{X}\right)$ and variances (*Var*(*X*)) or covariance (*Cov*(*X*)) of each parameter. The Resource Availability Model includes DIN:DIP and chlorophyll *a* concentration and the Full Model includes means and variances or covariances for all listed environmental parameters. Environmental data are the residuals from a regression between each parameter versus log(depth) and distance from shore. Bioerosion rates were square-root transformed to meet model assumptions. The ranges for each environmental parameter are included in Silbiger et al. 2014.(PDF)

S1 Dataset Dataset supporting this paper including raw secondary accretion and bioerosion data. Environmental data are available at http://doi.pangaea.de/10.1594/PANGAEA.846699.
(XLSX)

S1 Methods Detailed description of experimental design. (PDF)
