# Supplementary figures and images for: A Novel μCT Analysis Reveals Different Responses of Bioerosion and Secondary Accretion to Environmental Variability
Source: PLoS One. 2016 Apr 13;11(4):e0153058. doi: 10.1371/journal.pone.0153058 (PMC4830455; doi:10.1371/journal.pone.0153058)

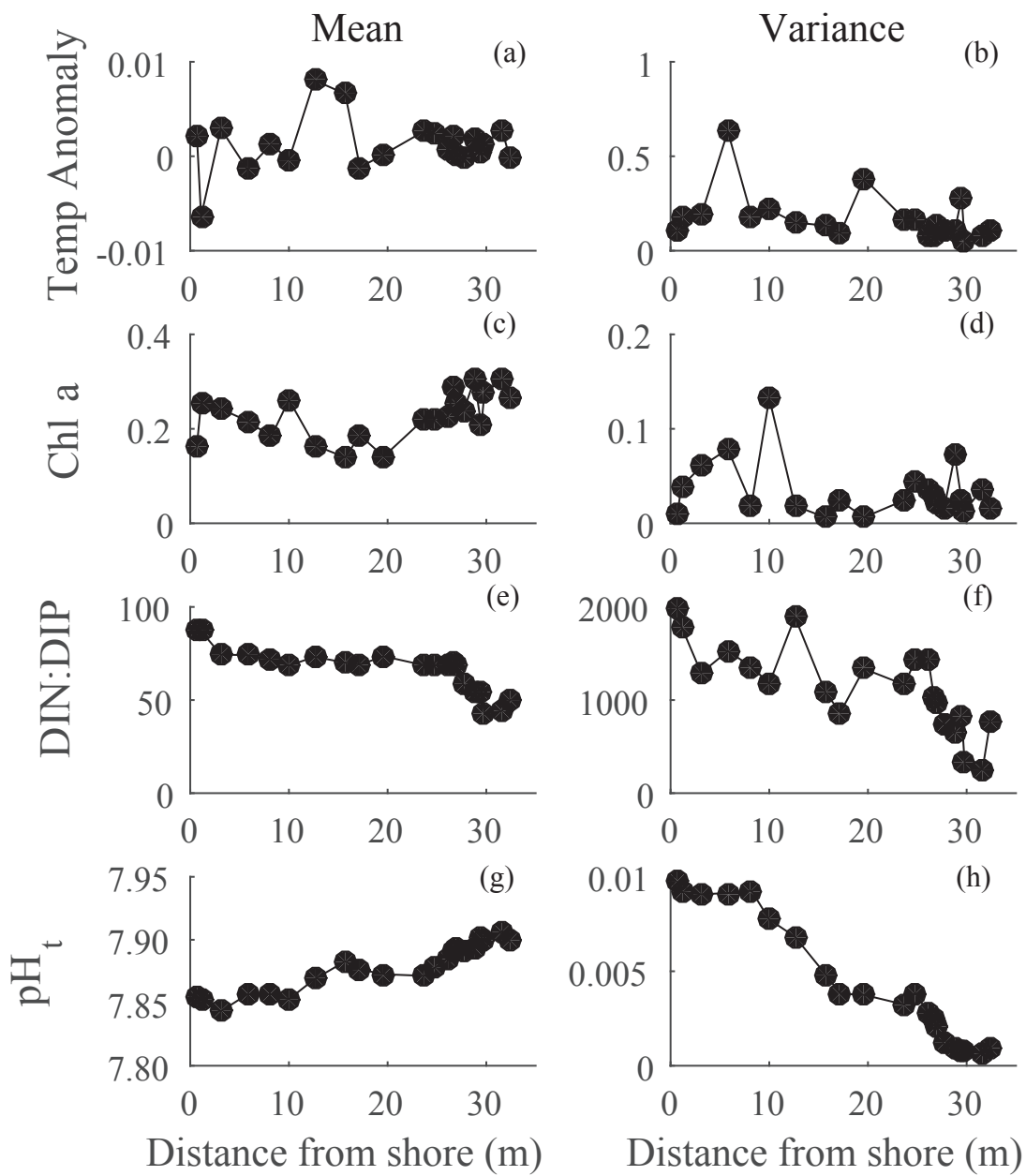

Supplement: S1 Fig — Means and variances for temperature anomalies (a-b), chlorophyll a (μg l−1) (c-d), DIN:DIP (e-f), and pHt (total scale) (g-h) along the transect (N = 21). (PDF) [file pone.0153058.s003.pdf]

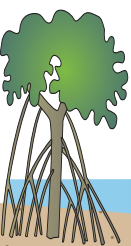

Reef Crest

Reef Slope

Mobile Sensor

24 meters

1 m

5 m

Mobile Sensor

8 meters

Permanent  
Sensor

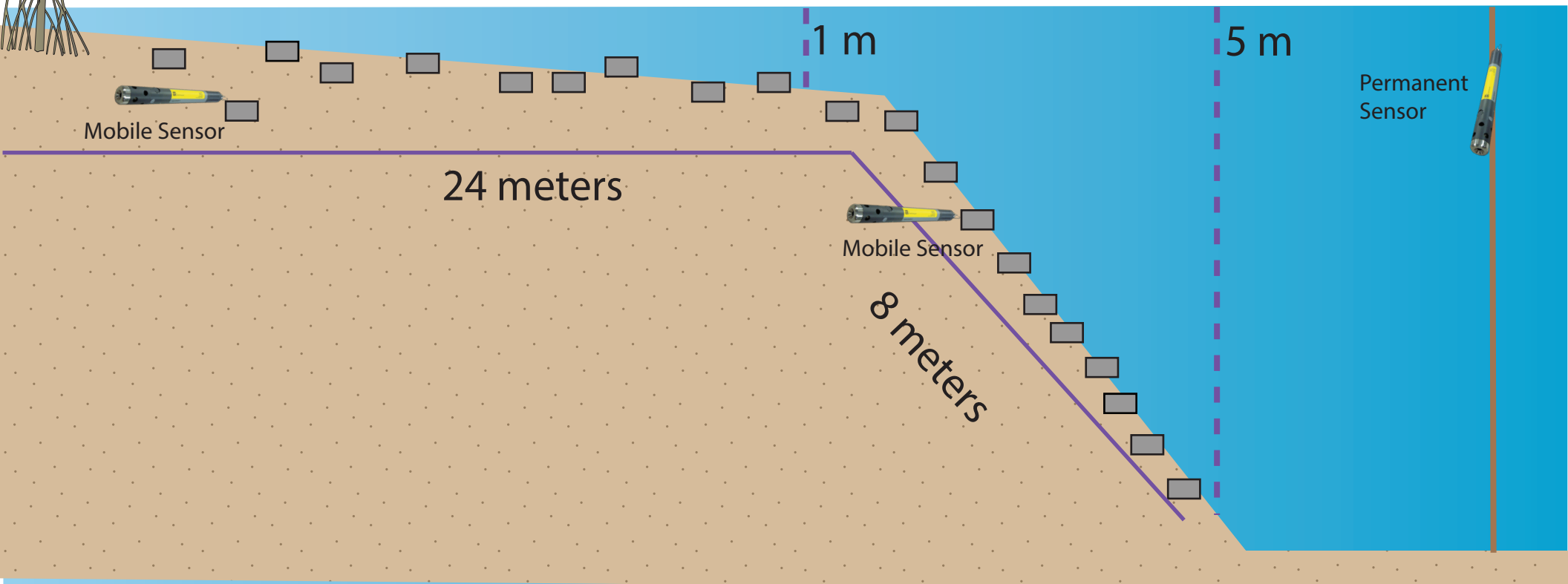

Supplement: S2 Fig — Experimental blocks (grey rectangles) were stratified between reef flat and reef slope along a 32 m transect and were deployed for one year. The average depth ranged from 0.5 to 4.5 m. Discrete environmental samples were collected directly above each experimental block. Continuous sensors were stationed over each block for a minimum of two weeks (mobile sensors) and were normalized to a continuous time series from a permanent sensor station (Permanent sensors). Picture of YSI Sonde is from sontek.com. (PDF) [file pone.0153058.s004.pdf]

Sqrt(Secondary Accretion Rate mm yr<sup>-1</sup>)

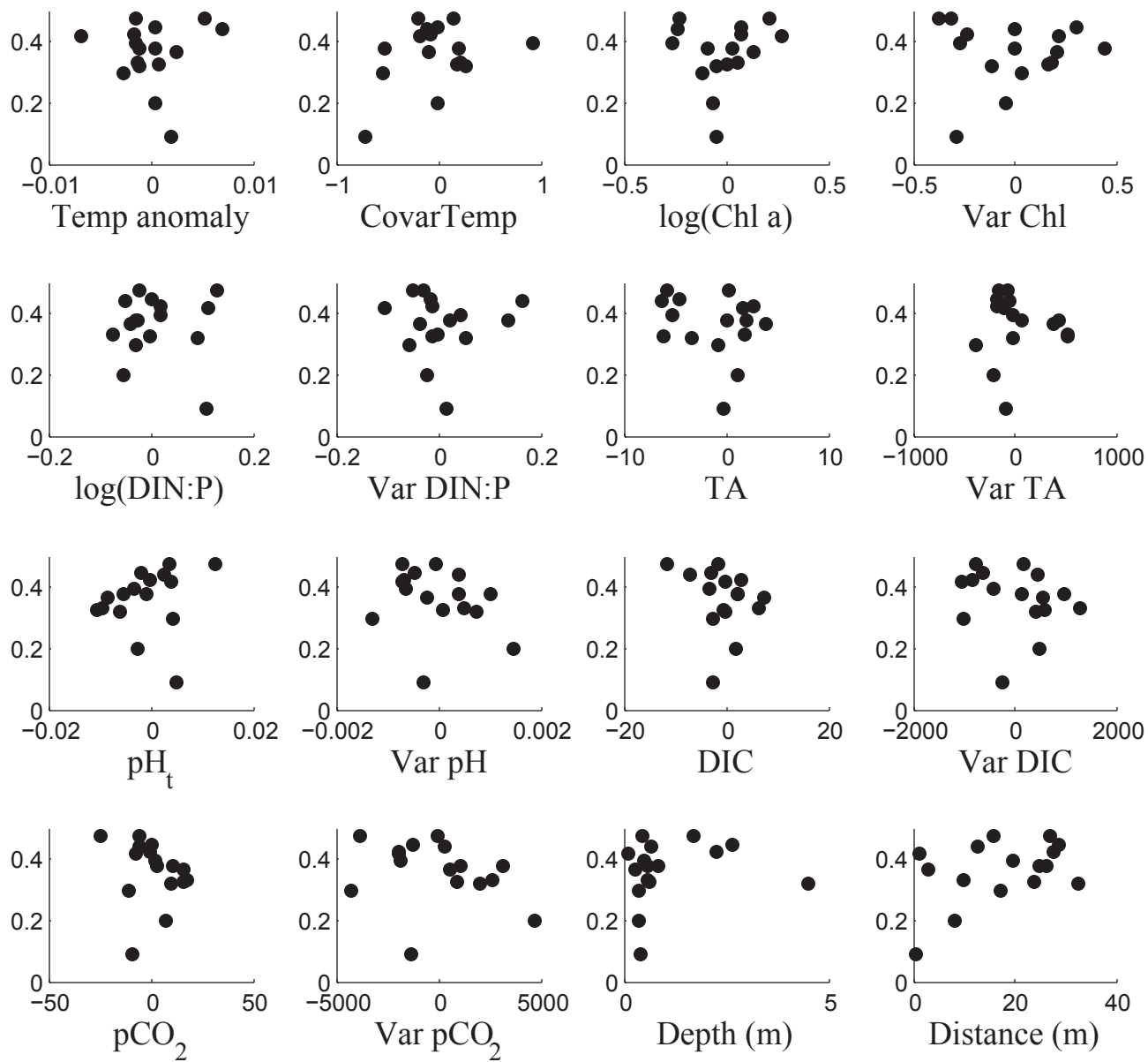

Supplement: S3 Fig — Environmental parameters were regressed against depth and distance from shore and the residuals from those regressions are used in this figure. (PDF) [file pone.0153058.s005.pdf]

Sqrt(Bioerosion Rate  $\text{kg m}^{-2} \text{yr}^{-1}$ )

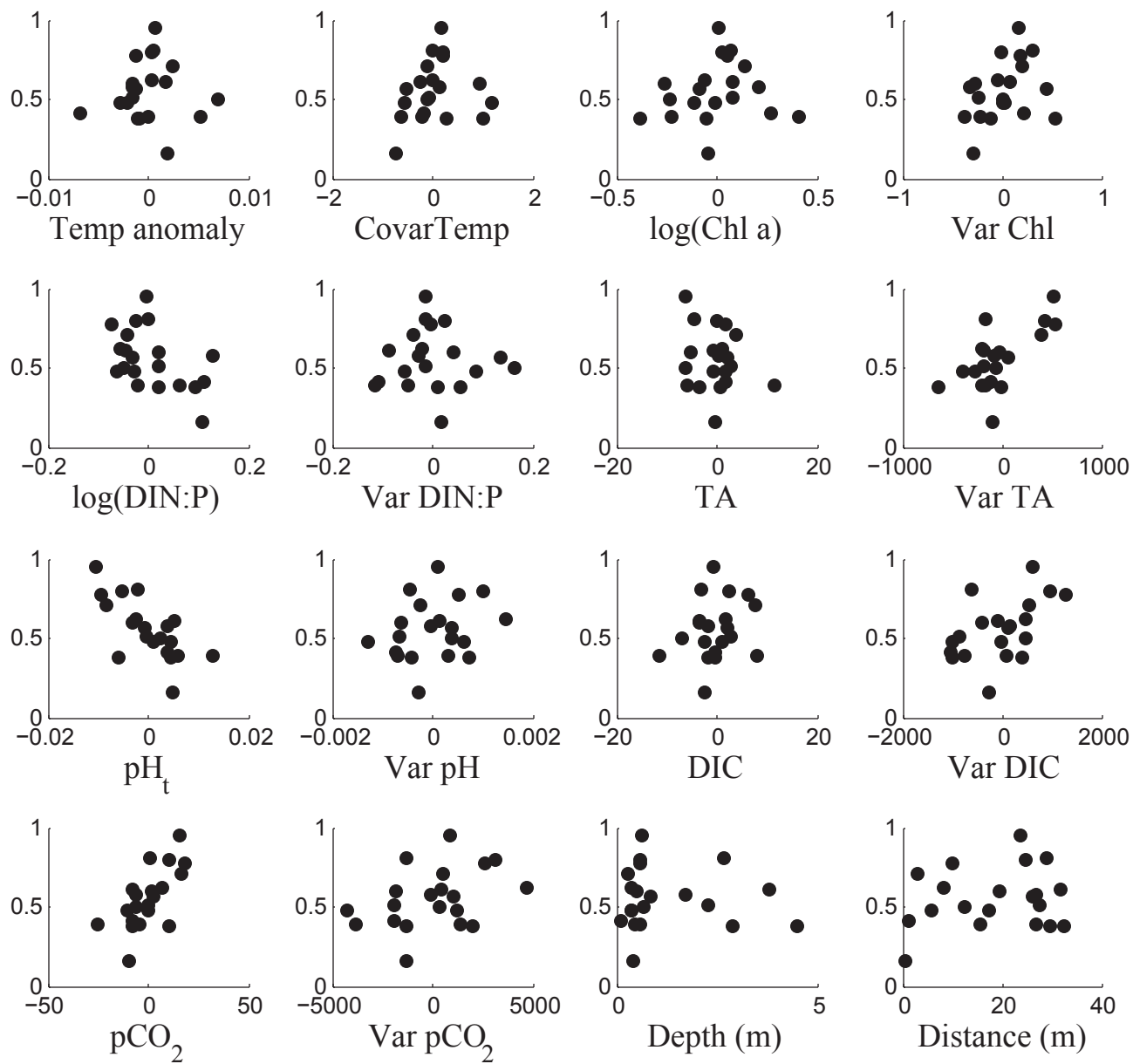

Supplement: S4 Fig — Environmental parameters were regressed against depth and distance from shore and the residuals from those regressions are used in this figure. (PDF) [file pone.0153058.s006.pdf]

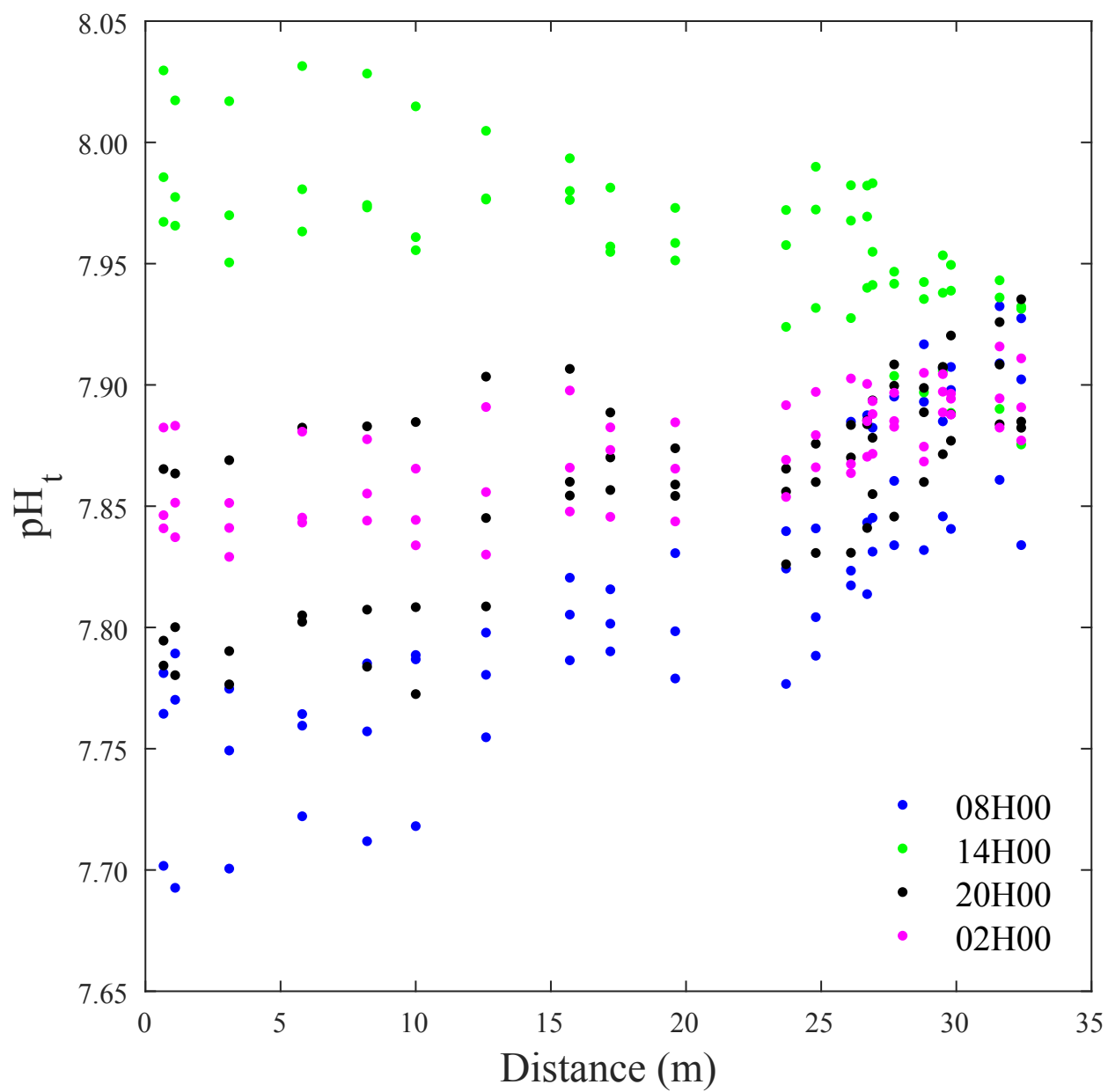

Supplement: S5 Fig — In each sampling period, water samples were collected at 08H00 (blue), 14H00 (green), 20H00 (black), and 02H00 (magenta), resulting in 12 samples at each of the 21 blocks. (PDF) [file pone.0153058.s007.pdf]

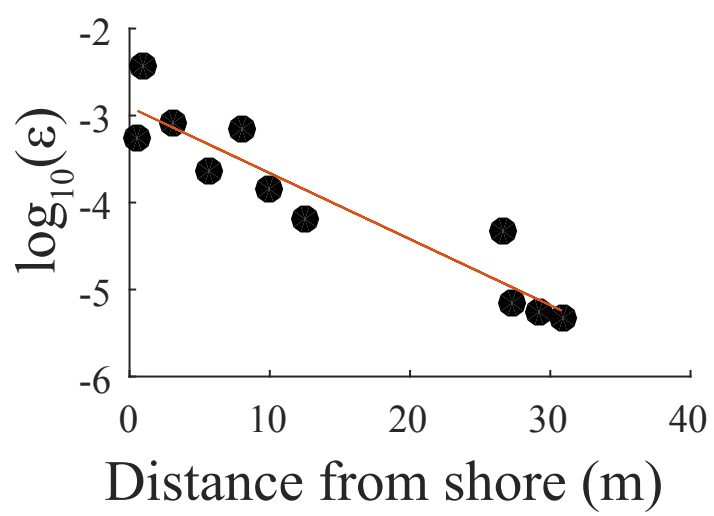

Supplement: S6 Fig — Turbulence was measured at 11 of the 21 sites and there was a significant relationship between ϵ and distance from shore (F11,9 = 63.1, p<0.0001, R2 = 0.88). (PDF) [file pone.0153058.s008.pdf]
