# Supplementary material for: A Novel μCT Analysis Reveals Different Responses of Bioerosion and Secondary Accretion to Environmental Variability
Source: PLoS One. 2016 Apr 13;11(4):e0153058. doi: 10.1371/journal.pone.0153058 (PMC4830455; doi:10.1371/journal.pone.0153058)
Supplement: S1 Table — k is the number of parameters in the model, -log(L) is the negative log likelihood of the model, AICc is the Akaike Information Criterion corrected, ΔAICc is the difference from the lowest AICc value, R2 is the proportion of total variance explained by the model, and Rank is the rank of the model with 1 representing the best fit. Each model is a linear regression of total bioerosion versus the means (X¯) and variances (Var(X)) or covariance (Cov(X)) of each parameter. The Resource Availability Model includes DIN:DIP and chlorophyll a concentration and the Full Model includes means and variances or covariances for all listed environmental parameters. Environmental data are the residuals from a regression between each parameter versus log(depth) and distance from shore. Bioerosion rates were square-root transformed to meet model assumptions. The ranges for each environmental parameter are included in Silbiger et al. 2014. (PDF) [file pone.0153058.s009.pdf]

S1 Table

S1 Table. Bioerosion Model Selection with all carbonate parameters.

	k	-log($\mathcal{L}$)	AICc	ΔAIC	R^2	Rank
**pH**	4.00	-12.42	-17.33	0.00	0.51	1
**pCO_2**	4.00	-11.76	-16.01	1.32	0.47	2
**TA**	4.00	-11.35	-15.20	2.13	0.45	3
**DIC**	4.00	-8.35	-9.21	8.12	0.26	4
**Distance**	3.00	-5.87	-7.04	10.29	0.04	5
**Depth**	3.00	-5.49	-6.27	11.06	0.004	6
**Resource Availability**	6.00	-9.79	-5.30	12.03	0.37	7
**Temperature**	4.00	-5.81	-4.12	13.21	0.04	8
**Full**	18.00	-45.27	249.47	266.80	0.99	9

k is the number of parameters in the model, -log($\mathcal{L}$) is the negative log likelihood of the model, AIC_c is the Akaike Information Criterion corrected, ΔAIC_c is the difference from the lowest AIC_c value, R^2 is the proportion of total variance explained by the model, and Rank is the rank of the model with 1 representing the best fit. Each model is a linear regression of total bioerosion versus the means ($\overline{X}$) and variances ($Var(X)$) or covariance ($Cov(X)$) of each parameter. The Resource Availability Model includes DIN:DIP and chlorophyll a concentration and the Full Model includes means and variances or covariances for all listed environmental parameters. Environmental data are the residuals from a regression between each parameter versus log(depth) and distance from shore. Bioerosion rates were square-root transformed to meet model assumptions. The ranges for each environmental parameter are included in Silbiger et al. 2014.
