# Supplementary material for: A Novel μCT Analysis Reveals Different Responses of Bioerosion and Secondary Accretion to Environmental Variability
Source: PLoS One. 2016 Apr 13;11(4):e0153058. doi: 10.1371/journal.pone.0153058 (PMC4830455; doi:10.1371/journal.pone.0153058)
Supplement: S1 Methods — (PDF) [file pone.0153058.s011.pdf]

Supporting Information

S1 Methods

Study site: Our study site is located in Kāneʻohe Bay, Oʻahu on the windward (eastern) side of Moku o Loʻe (Coconut Island), adjacent to the Hawaiʻi Institute of Marine Biology; N21°25.975', W157°47.175'). This fringing reef is dominated by *Porites compressa* and *Montipora capitata*, with occasional colonies of *Pocillopora damicornis, Fungia scutaria,* and *Porites lobata.* Kāneʻohe Bay is a protected, semi-enclosed embayment; the water residence time can be >1 month in the protected southern portion of Kāneʻohe Bay, where our study was located [1]. Wave action is low [1–3], and currents are slow (5cm s^{-1} maximum) and tidally driven (mean and maximum tidal ranges are 0.7 and 1.1m, respectively) [1,3]. Daily averages in pH, temperature, and O_2 in the Kāneʻohe Bay waters just offshore our site ranged from 7.83 - 8.03, 21.84-27.86 °C, and 5.82-7.81 mg L^{-1}, respectively, during our study period [4].

Environmental Parameters: The discrete water samples were collected directly above each block within two days of spring tide at 08:00, 14:00, 20:00, and 02:00 on September 10-11, 2011, December 12-13, 2011, and April 4-5, 2012. All discrete water samples were collected on snorkel or SCUBA using 60 and 120ml plastic syringes. Syringes and storage vials were all pre-cleaned in a 10% HCl bath for 24 hours and rinsed three times with MilliQ water; during sample collection and processing syringes were rinsed three times with sample water. The environment was sampled more continuously for temperature and depth (sampling rate of 0.1 min^{-1}) using one permanent and two mobile monitoring stations. Two mobile stations were deployed at a time, one on the reef flat and one on the reef slope, to get simultaneous measurements at two different blocks on the transect. Mobile stations (Sonde 600XLM, YSI Incorporated) were positioned 5 - 10cm above each block for a two-week period between May 2011 and March 2012. Blocks were sampled in random order, ensuring that the spatial gradient along the transect was not systematically confounded by temporal trends or seasonality [4]. The permanent monitoring station (Sonde 6600-V2-4, YSI) was mounted to a pole a few meters away from the transect, downward facing at 1.7m depth over a 3m deep bottom, with sensors for temperature, depth, conductivity, pH, and O_2 to characterize the background water column conditions for the duration of the experiment. All multi-parametric probes were calibrated periodically using standard procedures and calibration solutions. The permanent station was recovered, cleaned, calibrated, and re-deployed 3 times during the study, and the mobile station probes were calibrated 7 times. Pre-calibration measurements of commercial standard solutions were conducted to detect sensor drift, although none was found for the period of study. Environmental data from the transect are reported in Silbiger et al. (2014) [5] and background water column data are reported in Guadayol et al. (2014) [4].

Nutrients and Chlorophyll: Water samples collected for nutrients were immediately filtered through combusted 25 mm diameter glass fiber filters (GF/F 0.2 µm) and transferred into 50 ml plastic centrifuge tubes. Nutrient samples were frozen and later analyzed for NO_3^-, NO_2^-, NH_4^+, and PO_4^{3-} on a Seal Analytical AA3 HR Nutrient Analyzer at the UH SOEST Laboratory for Analytical Chemistry. GF/F filters were folded in half, wrapped in aluminum foil, and frozen for chlorophyll *a* analysis using a

Turner Designs 10AU Benchtop Fluorometer. The ratio of dissolved inorganic nitrogen to dissolved inorganic phosphate (DIN:DIP) was used as a proxy for resource quality available to filter feeders [6], assuming that elemental composition of planktonic prey will be influenced by elemental composition of the water column, and was calculated from $([NO_3^-] + [NO_2^-] + [NH_4^+]):[PO_4^{3-}])$.

pH and TA Mean and variance in pH at each block was calculated from water samples along the transect. Water samples for pH were immediately transferred into 25 ml borosilicate glass vials, brought to a constant temperature of 25°C in a water bath, and immediately analyzed using an m-cresol dye addition spectrophotometric technique and calibrated against a Tris buffer of known pH from the Dickson Laboratory at Scripps Institution of Oceanography. TA was fixed with 100 μL of $HgCl_2$ and analyzed using open cell potentiometric titrations on a Mettler T50 autotitrator and calibrated against a Certified Reference Material following Dickson et al. (2007) [7] protocols. *In situ* pH and all other carbonate parameters were estimated using CO2SYS [8] with the following parameters: pH_t, TA, temperature, and salinity. The K1K2 dissociation constants were from Mehrbach (1973) [9] (refit by Dickson and Millero (1987) [10]) and HSO_4^- dissociation constants were taken from Uppstrom (1974) [11] and Dickson (1990) [12]. Accuracy for TA and pH was better than 0.8% and 0.04%, respectively, and the precision was 3.55 µEq and 0.004 pH units.

Temperature: Temperature sensors (YSI 6560) were thermistors with manufacturer-reported accuracy of ±0.15°C and resolution of 0.01°C (YSI Incorporated 2011). Average differences in temperature along the transect were small and measured as a relative anomaly from the permanent station: $((\bar{x}_{mobile} - \bar{x}_{permanent})/\bar{x}_{permanent})$. To measure relative variability in temperature across the transect, we calculated the covariance in temperature between the mobile and permanent sensor arrays over a two-week period and compared this covariance across the transect.

Depth and Distance from Shore: Depth is the average depth measured at each block over the two week deployment of the mobile station. Distance from shore is the along-transect distance.

Turbulent Kinetic Energy Dissipation Rate (ϵ): Water velocity data were obtained with acoustic doppler velocimeters (Vectrino Field, Nortek A.S.) which were deployed 5-10 cm above the blocks along with the multiparametric sondes measuring temperature and depth at 11 of the 21 stations. Unfortunately one of the velocimeters broke during deployment, and flow data could not be acquired for the rest of the stations and therefore was not included in the model selection. However, given the tight correlation between ϵ and distance from shore ($R^2 = 0.88$), distance from shore was used as a proxy for the hydrodynamic gradient.

Spikes were removed following a 3D phase-space thresholding technique [13, 14] in the beam coordinates. Values with correlations <60 were also removed [15]. Gaps were linearly interpolated when shorter than 10 measurements. Empirical orthogonal functions (EOF) were used to align coordinates to streamwise/cross-stream axes for the entire sampling period

Turbulent kinetic energy dissipation rates (ϵ; $m^2 s^{-3}$) were estimated using the inertial subrange dissipation method [16]. Briefly, data was partitioned in 10 minutes intervals, the same sampling period as the multiparameter Sonde measuring temperature and depth. For each segment, data was further partitioned into 180 second segments of uninterrupted data, from which the fast Fourier transforms were obtained. A smoothed spectra was generated by averaging all the raw spectra. The inertial

subrange was identified in the log transformed spectra as the segment that best fit a -5/3 model, with a minimum coefficient of determination (R^2) of 0.75, and encompassing at least one order of magnitude of frequencies. Fits were evaluated using F statistics and R^2. To account for the effect of advection by current and waves on the turbulent spectra, we used a generalized frozen turbulence model [17]. All calculations were done using MATLAB.

References

1. Lowe RJ, Falter JL, Monismith SG, Atkinson MJ (2009) A numerical study of circulation in a coastal reef-lagoon system. Journal of Geophysical Research 114: C06022.
2. Smith SV, Kimmerer WJ, Laws EA, Brock RE, Walsh TW (1981) Kaneohe Bay sewage diversion experiment- perspectives on ecosystem responses to nutritional perturbation. Pacific Science 35: 279-402.
3. Lowe RJ, Falter JL, Monismith SG, Atkinson MJ (2009) Wave-driven circulation of a coastal reef-lagoon system. Journal of Physical Oceanography 39: 873–893.
4. Guadayol Ò, Silbiger NJ, Donahue MJ, Thomas FI (2014) Patterns in temporal variability of temperature, oxygen and pH along an environmental gradient in a coral reef. PLoS One 9: e85213.
5. Silbiger NJ, Guadayol Ò, Thomas FI, Donahue MJ (2014) Reefs shift from net accretion to net erosion along a natural environmental gradient. Marine Ecology Progress Series 515: 33–44.
6. Hauss H, Franz J, Sommer U (2012) Changes in N: P stoichiometry influence taxonomic composition and nutritional quality of phytoplankton in the Peruvian upwelling. Journal of Sea Research 73: 74-85.
7. Dickson A, Sabine C, Christian JR (2007) Guide to Best Practices for Ocean CO_2 Measurements. PICES Special Publication 3.
8. van Heuven S, Pierrot D, Rae J, Lewis E, Wallace D (2011) MATLAB Program Developed for CO2 System Calculations. ORNL/CDIAC-105b. Carbon Dioxide Information Analysis Center, Oak Ridge National Laboratory, U.S. Department of Energy, Oak Ridge, Tennessee. doi:$10.3334/CDIAC/otg.CO2SYS~\_~MATLAB~\_~v1.1$.
9. Mehrbach C (1973) Measurement of the apparent dissociation constants of carbonic acid in seawater at atmospheric pressure. Limnology and Oceanography 18: 897-907.
10. Dickson A, Millero F (1987) A comparison of the equilibrium constants for the dissociation of carbonic acid in seawater media. Deep Sea Research Part A Oceanographic Research Papers 34: 1733–1743.
11. Uppström LR (1974) The boron/chlorinity ratio of deep-sea water from the pacific ocean. In: Deep Sea Research and Oceanographic Abstracts. volume 21, pp. 161–162.
12. Dickson AG (1990) Standard potential of the reaction:$AgCl(s) + 12H2(g) = Ag(s) + HCl(aq)$, and and the standard acidity constant of the ion HSO_4^- in synthetic sea water from 273.15 to 318.15 k. The Journal of Chemical Thermodynamics 22: 113–127.

13. Goring DG, Nikora VI (2002) Despiking acoustic doppler velocimeter data. Journal of Hydraulic Engineering 128: 117–126.

14. Wahl TL (2003) Discussion of "Despiking acoustic doppler velocimeter data" by Derek G. Goring and Vladimir I. Nikora. Journal of Hydraulic Engineering 129: 484–487.

15. McLelland SJ, Nicholas AP (2000) A new method for evaluating errors in high-frequency ADV measurements. Hydrological Processes 14: 351–366.

16. Bluteau CE, Jones NL, Ivey GN (2011) Estimating turbulent kinetic energy dissipation using the inertial subrange method in environmental flows. Limnology and Oceanography: Methods 9: 302–321.

17. Lumley J, Terray E (1983) Kinematics of turbulence convected by a random wave field. Journal of Physical Oceanography 13: 2000–2007.
